# fNIRS-derived neurocognitive ratio as a biomarker for neuropsychiatric diseases

**DOI:** 10.1117/1.NPh.8.3.035008

**Published:** 2021-09-30

**Authors:** Ata Akın

**Affiliations:** Acibadem University, Department of Medical Engineering, Ataşehir, Istanbul, Turkey

**Keywords:** fNIRS, neuropsychiatric diseases, functional connectivity, global efficiency, cognitive quotient, neurocognitive ratio

## Abstract

**Significance:** Clinical use of fNIRS-derived features has always suffered low sensitivity and specificity due to signal contamination from background systemic physiological fluctuations. We provide an algorithm to extract cognition-related features by eliminating the effect of background signal contamination, hence improving the classification accuracy.

**Aim:** The aim in this study is to investigate the classification accuracy of an fNIRS-derived biomarker based on global efficiency (GE). To this end, fNIRS data were collected during a computerized Stroop task from healthy controls and patients with migraine, obsessive compulsive disorder, and schizophrenia.

**Approach:** Functional connectivity (FC) maps were computed from [HbO] time series data for neutral (N), congruent (C), and incongruent (I) stimuli using the partial correlation approach. Reconstruction of FC matrices with optimal choice of principal components yielded two independent networks: cognitive mode network (CM) and default mode network (DM).

**Results:** GE values computed for each FC matrix after applying principal component analysis (PCA) yielded strong statistical significance leading to a higher specificity and accuracy. A new index, neurocognitive ratio (NCR), was computed by multiplying the cognitive quotients (CQ) and ratio of GE of CM to GE of DM. When mean values of NCR ($\overset{¯}{NCR}$) over all stimuli were computed, they showed high sensitivity (100%), specificity (95.5%), and accuracy (96.3%) for all subjects groups.

**Conclusions:**$\overset{¯}{NCR}$ can reliable be used as a biomarker to improve the classification of healthy to neuropsychiatric patients.

## 1 Introduction

Although fNIRS has been around over 30 years now, its clinical efficacy and role are still being questioned due to its low specificity and sensitivity, especially in the area of neuropsychiatric diseases. Many researchers have been trying to improve its efficacy, sensitivity, and specificity in clinical settings by either improving its technology or the post processing analysis methods. Over the last 20 years, the richness of fNIRS data due to its ease and speed of data collection, non-invasiveness and access to local activity have become even more attractive to cognitive neuroscientists in testing multitude of data processing and neuroscientific hypotheses. Strangely though, the brain does not work locally.^1^^–^^4^

fNIRSians have long been in search of a killer application that would secure the place of fNIRS in clinical settings. To this end, fNIRS have been applied to subjects of all ages and health conditions.^5^^–^^8^ Discussions regarding the limitations and ways to overcome these are a few yet they all have helped us redirect our efforts in proposing ever more innovative solutions. There are good reviews on the promise of fNIRS in neuropsychiatry.^5^^,^^7^^,^^9^^–^^14^

So far, fNIRS researchers have focused more on the data analytics side than developing of novel technologies. We have enjoyed the availability of various fNIRS systems but at the cost of standardization of probe designs, data collection methodologies, and even more on the data analytics.^13^ The lack of standardization on these issues has made it increasingly more difficult to compare the findings from different studies.^15^ Moreover, physics of photon migration through the layers of the head limits the specificity of the fNIRS device to cortical layers. Collected data become an amalgam of physiological activity from each layer the photon interacts with. Hence the data are known to be contaminated with background systemic physiological fluctuations that are undoubtedly correlated with the cognitive activity.^16^^,^^17^ The low specificity of the CW-fNIRS devices can be overcome with time resolved systems albeit at a greater cost and complications of data collection. Many novel data analysis methods have been proposed to extract the brain originated, task related data from the collected data.^18^^–^^20^ Still there is no consensus on how to approach the fNIRS data, leading to the unsettling yet quite accurate prediction of Drs. Quaresima and Ferrari: “The prediction of the future directions of fNIRS for assessing brain function during human behavior in natural and social situations is not easy.”^13^ It is, hence, only logical to propose an analysis method (a pipeline of data analysis) that would avoid the pitfalls of the standardization issues faced in fNIRS signal processing field. The following Table 1 is a list of minimum hardware, data collection, and analysis recommendations for fNIRS-based cognitive research that are derived from experience and literature:

**Table 1:** Recommendations for fNIRS based research.

Hardware requirements	• Number of channels ≥8
	• Collect data from both hemispheres
	• Sampling rate ≥0.5 Hz per channel
	• Number of wavelengths ≥2
Task requirements	• Duration of collected data ≥10 min
	• Block stimulus with block duration ≥20 s
	• Stimulus type ≥2
	• Resting data ≈30 s
Analysis requirements	• Prefer [HbO] data
	• Avoid single channel data analysis
	• Prefer a dimensionless analysis (i.e., normalization of data)
	• Prefer a multichannel analysis (i.e., FC)
	• Prefer a metric that summarizes and captures the behavior of multichannel data (i.e., FC strength such as *GE*.)

This study proposes an improved post-processing approach to data obtained from fNIRS recordings over our previous paper.^21^ The sole aim was to converge on a data analysis pipeline that will be accepted and adapted easily by fellow fNIRSians. The proposed algorithm should have a common denominator, a base for anyone to build upon. The algorithm aims to improve the statistical significance of the fNIRS findings, and hence, the trust on the system. The major aim is to boost the statistical significance of the GE values; hence, the accuracy of classification of fNIRS findings in a set clinical data obtained in our group’s previous studies.

## 2 Methods and Materials

### 2.1 Subjects and Experimental Procedure

About 13 healthy subjects (six female) at an average age of 26, 20 patients with migraine without aura (12 female) at an average age of 27, 26 patients with obsessive compulsive disorder (11 female) at an average age of 29, and 21 schizophrenia patients (10 female) at an average age of 28 participated in this study. The study protocol was approved by the Ethics Committee of Pamukkale University in 2008. Parts of these data were published by our group and coworkers.^22^^–^^30^ Consents were obtained from all subjects and they were all informed about the study before the experiment. Subjects were seated in a dimly illuminated insulated room and they were told to look at a computer screen placed in front of them.

Subjects responded to the computerized color word matching Stroop task that involved three sets of stimuli: neutral (N), congruent (C), and incongruent (I) stimuli. The task involved 15 N, 15 C, and 15 I stimuli presented in blocks of five sequential stimuli. The inter stimulus interval was 4 s. The rest between each block was 20 s. The stimuli blocks were randomized for each subject. The subject was asked to respond with left or right mouse click depending on whether the stimulus was a match or not. The task started with a 30 s of rest and ended with a 30 s of rest.^24^^,^^31^

### 2.2 fNIRS Equipment

**Fig. 1:**
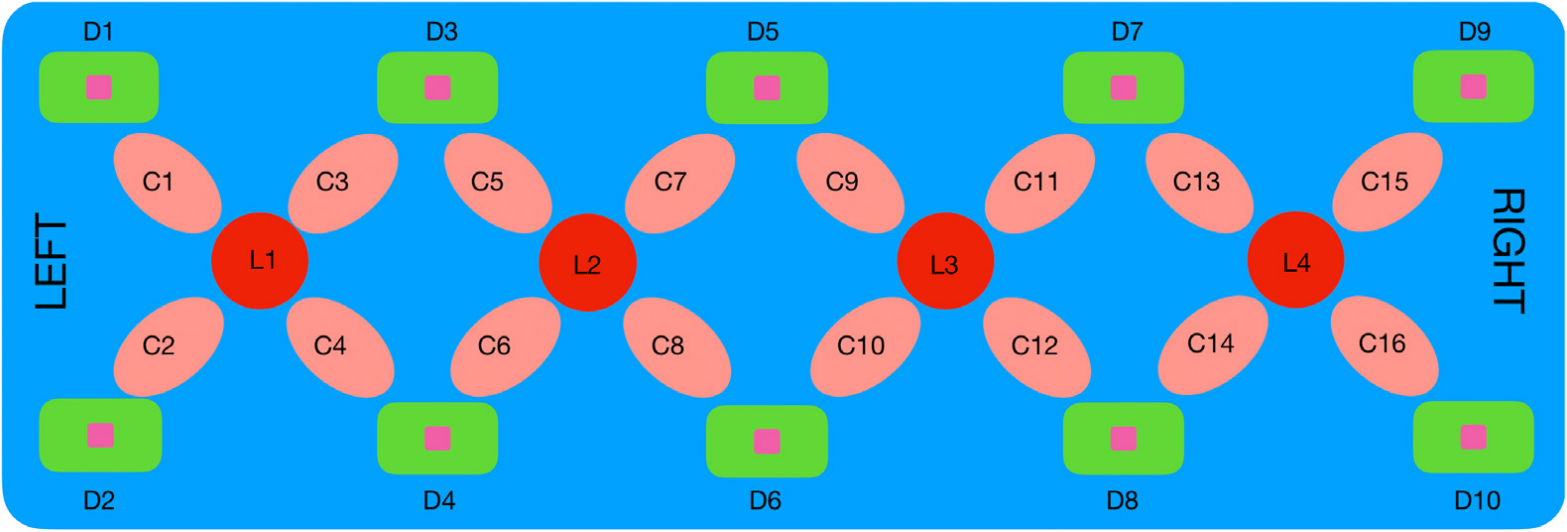
Rectangular probe geometry of the fNIRS NIROXCOPE 301. $L_{i}$ are the LEDs; $D_{i}$, the detectors; and $C_{i}$, the channels.

The fNIRS system (NIROXCOPE 301) was developed at the Neuro-Optical Imaging Laboratory of Bogazici University.^23^^,^^30^ NIROXCOPE 301 has a sampling frequency of 1.77 Hz, and it consists of a data acquisition unit, a data collecting computer, and a flexible probe to place on the forehead of the subjects. The probe has a rectangular design housing four dual wavelength light-emitting diodes (LED) emitting at 730 and 850 nm. Each LED ($L_{i}$, i=1…4) is surrounded by four detectors ($D_{i}$, i=1…10) placed 2.5 cm away from the center of an LED as seen in Fig. 1. A channel is a pair of LED and detector that surrounds that LED. Since several of the in-between detectors are shared, there are 16 channels ($C_{i}$ i=1…16). Since the received light intensity is inversely proportional to the square of the distance between a source and a detector, data from long range channel pairs were not collected (i.e., between $L_{1}$ and $D_{5}$, $D_{7}$, or $D_{9}$).

The validity of this probe design and its ability to detect brain tissue were discussed in our previous study^18^ as well as its efficacy in providing cognition-related signals.^21^^,^^24^^–^^26^^,^^30^^,^^32^

### 2.3 Analysis of the fNIRS Data

**Fig. 2:**
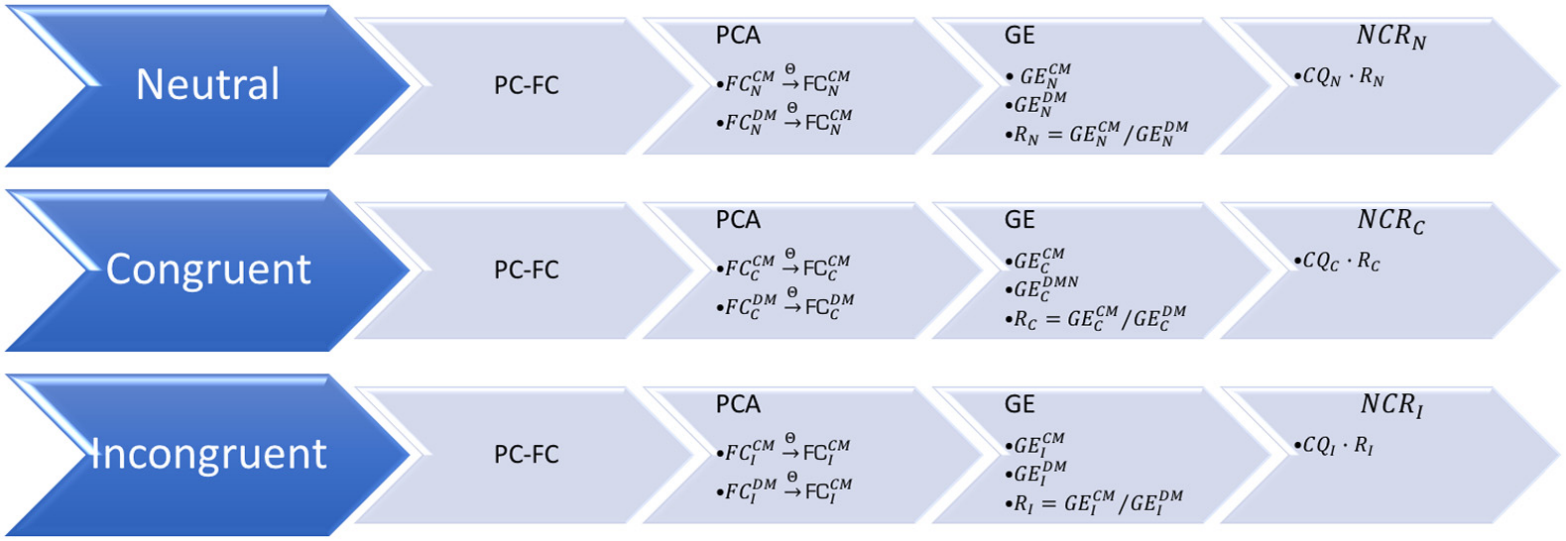
Block diagram of the NCR algorithm (abbreviations are given in the text).

fNIRS data are known to be contaminated with systemic background fluctuations. So before attempting to generate connectivity matrices from pair-wise correlations, one should try to minimize the effect of this background fluctuation so that the effect of this dominant signal is eliminated. Assuming that any correlation between two channels will be dominated by this common background signal, our previous study aimed to show that a partial correlation (PC)-based analysis will yield a less biased insight into the underlying connectivity due to task. In that paper, an outline of the signal processing steps were given in detail.^21^ As a quick summary, a signal processing pipeline was developed to compute the FC matrices (FC) from [HbO] signals by using a PC method, rather than the conventional pearson correlation analysis. Then these matrices were used to compute the GE values. In this paper, an additional step in between the FC matrices and GE computation is proposed by employing the principal component analysis (PCA). As a last step, a new biomarker as a function of behavioral and fNIRS deriven features: neurocognitive ratio (NCR). The details of the derivation and computation of this biomarker is explained in Sec. 2.4 and the block diagram of the algorithm is shown in Fig. 2.

This paper will present the results of this PCA-based FC analysis, hence called the FC-(PC)2: FC analysis via PCA based PC.

#### 2.3.1 Preparation of fNIRS data for FC analysis

**Fig. 3:**
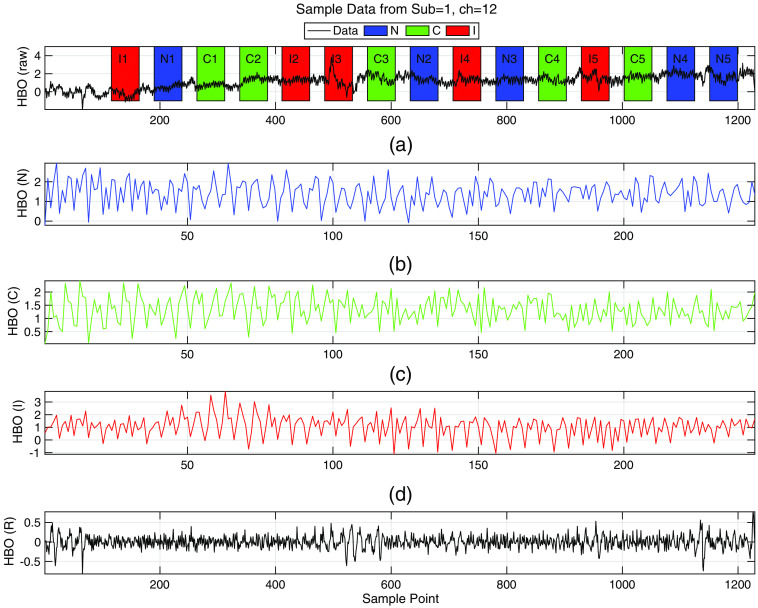
Preparation steps shown on a sample [HbO] data. (a) Raw and unprocessed [HbO] data of subject 3 (control), from channel 12, where the patched regions designate the stimuli blocks. (b) Concatenated [HbO] data for N stimulus where segments of N data ($N_{i}$, i=1…5) from the original data are concatenated sequentially to create the [HbO] (N) time series data, (c) Concatenated [HbO] (C) time series data for C stimulus, (d) Concatenated [HbO] (I) time series data for I stimulus, and (e) the regressor computed as explained in Sec. 2.3.2.

FC is a method where correlations from time series data are used to create a matrix called the functional connectivity matrix (FC). The matrix is a N×N matrix, where N is the number of channels for fNIRS (number of voxels for fMRI). In this study, N=16. Since the study protocol involved the Stroop task with three types of stimuli, it was reasonable to create 3FCs. fNIRS data that will be fed into FC calculations were prepared in the following steps to generate these matrices:

1. Locate the stimuli blocks in fNIRS time signal for each subject [i.e., Fig. 3(a)]
2. Create three concatenated stimulus time series for each channel [i.e., Figs. 3(b) and 3(d)]
3. Generate the regressor to be used in PC calculations as explained in Sec. 2.3.2, [i.e., Fig. 3(e)].

#### 2.3.2 Functional connectivity via PC

PC provides a cleaner (or less biased) relationship between two variables after removing a common effect present in both of the variables. The PC coefficient ($r_{i,j|k}$) between any two channels (i,j) in the presence of a common influencer (k) is computed as follows:^33^

$$r_{i,j|k}=\frac{r_{i,j}-r_{i,k}r_{j,k}}{\sqrt{\left(1-r_{i,k}^{2}\right)}\sqrt{(1-r_{j,k}^{2}})}.$$ (1)

[HbO] data from each channel are passed through a high pass filter (butterworth, eigth order, cutoff at $f_{c}=0.09\;\mathrm{Hz}$, stop-band at $f_{s}=0.1\;\mathrm{Hz}$) to obtain the $HBO_{H}^{i}$. The regressor used in PC-based FC analysis is obtained by averaging this signal over all the channels. Hence ${\overset{¯}{HBO}}_{R}=\underset{i}{\sum }HBO_{H}^{i}$ is used to regress out the systemic physiological affects from the correlation of the unprocessed [HbO] signals from two channels. Once the regressor ${\bar{HBO}}_{R}$ is computed, individual regressors for N, C, and I stimuli are generated similar to the concatenation explained in Fig. 3. Then these regressors are used in the computations of the PC coefficients as entries to the FC. This analysis is performed for each subject. At the end of this part of this analysis, 3FCs are generated from each subject’s fNIRS. The FC matrices computed for stimulus based time series are thus termed $FC_{N}$, $FC_{C}$, and $FC_{I}$.

#### 2.3.3 Functional connectivity via PCA based PC

In a review by Du et al.,^34^ it is claimed that statistical significance of the FC derived features (i.e., GE) can be improved by adding PCA after the FCs are calculated. Once an FC was generated, PCA was applied to the it. Since the matrices were 16×16, there were 16 PCs. The assumption in applying PCA to FC is the following:

$$FC_{i}=FC_{i}^{CM}+FC_{i}^{DM},\;i=N,C,I,$$ (2)

where $FC_{i}^{DM}$ is the FC matrix of the default mode network, and $FC_{i}^{CM}$ is the matrix for the cognitive mode network. Since these two matrices can be assumed to be linearly independent, PCA can be applied to separate them into their independent parts. The choice of PCs turned out to affect the statistical significance of the $GE^{CM}$ values computed after new FCs were reconstructed from the chosen PCs. The expectation is the convergence to a subset of PCs that will yield the strongest significance for $GE_{i}^{CM}$ (as explained in Sec. 2.3.4) while no significance for the $GE_{i}^{DM}$.^35^^,^^36^ A combinatorial search analysis was performed to find the best principal components to reconstruct the new $FC_{i}^{CM}$. The choice of how many principal components to be used was based mostly on the strongest PCA eigenvalues. However, components with lower strengths were also included in some cases. Once the best PC subset was found, this subset was used to resonstruct the $FC_{i}^{CM}$. Remaining PCs were then used to reconstruct the $FC_{i}^{DM}$, i=N,C,I. The expectation from these $FC_{i}^{DM}$ matrices is such that the t-statistics of the $GE_{i}^{DM}$, i=N,C,I will be low (no statistical significance). So the algorithm works as an optimization approach where the goal was to maximize the t-statistics (minimize the p value) of the $GE_{i}^{CM}$, i=N,C,I.

#### 2.3.4 Global efficiency

Global efficiency (GE) is one of the many metrics of graph-based network analysis and has been used in brain connectivity studies.^21^^,^^37^^–^^39^ This approach is intrinsic to cognitive neuroscience where the aim is to investigate the neural correlates of cognition.^40^^–^^42^ Several groups, including ours have reported that GE can be reliably used as a metric to quantify the information sharing efficiency of the $FC_{i}^{CM,DM}s$. In this analysis, channels can be considered as a set of vertices V and the PC coefficients as assigned weights on the set of edges E, between vertices to construct an undirected complete weighted graph G=(V,E).^43^^–^^45^

GE can be evaluated for a wide range of networks, including weighted graphs.^45^ Maximal possible GE occurs when all edges are present in the network. The *GE* value was computed by using the formulation of Latora and Marchiori’s:^46^

$$GE=\frac{1}{N(N-1)}\underset{i\neq j\in G}{\sum }\frac{1}{d_{ij}},$$ (3)

where $d_{ij}$ is defined as the smallest sum of the physical distances throughout all the possible paths in the graph from i to j.^46^ This equation requires the use of binary matrix entries. So, since there was always some sort of a connection between channels (the entries were never 0) a threshold had to be used to eliminate very low connections. So choosing an appropriate threshold value was necessary to convert the FCs to binary matrices:

$$\mathrm{FC}(i,j)=\{\begin{array}{ll} 1 & \text{if}\;|\mathrm{FC}(i,j)|>\Theta \\ 0 & \text{otherwise} \end{array},$$ (4)

where FC is a binarized matrix after a hard thresholding at the value Θ is applied to the |FC|. Here, Θ is not an actual correlation value, rather the number of highest correlation values to be kept in the matrix. It is worth noting that absolute values of the FC were used in this equation. This threshold value determines the number of non-zero nodes to be kept in the binarized matrix, which in turn effects the computation of the *GE* values.

In this analysis, two specific Θ node values were determined iteratively for each subject group (see Table 2 for group dependent Θ values), one for CM ($\Theta^{CM}$) and one for DM ($\Theta^{DM}$). A review on the choice of such a threshold (Θ) yielded a value of the highest 10% to 20% of all the entries in the |FC|s. A sweep of the best group-wise Θ that provided the highest statistical significance (lowest p value as shown in Table 2) for $GE^{CM}s$ for three types of stimuli yielded specific $\Theta^{CM}$ values for different subject groups. In contrast, $\Theta^{DM}$ was chosen for the highest p value for three stimuli types. The computation of the GE values by Eq. (3) was performed by the efficiency.m code from the Brain Connectivity Toolbox.^4^

**Table 2:** PCA components for subject groups that yielded the best p value for $GE^{CM}$.

Group	CM	DM	$\Theta^{CM}$	$\Theta^{DM}$	p value
Controls	[1⋯9]	Remaining	43	32	0.033
Migraine	[2, 3, 6, 7, 9, 16]	Remaining	40	28	0.0017
OCD	[1⋯12,14,16]	Remaining	38	48	0.02
Schizo	[1⋯5,6⋯9,10,12⋯16]	Remaining	27	41	0.048

### 2.4 Neurocognitive Ratio

Cognitive quotient (CQ) can be considered as a measure of level of cognitive effort exerted to fulfill a task. There are several indices, quotients, and metrics proposed to assess this effort. Usually these are in the form of combinations of various neuropsychological task scores^47^^–^^49^ and sometimes in the form of physiological parameters.^50^ Cognitive load is a similar concept and many physiological measures have also been proposed to quantify this effort.^51^ The assumption for using physiological measures to quantify cognitive load is that brain, just like a muscle, has to execute some sort of a physiological activity (preferably measurable) for a specific cognitive task.^52^ So, the holy grail of neuroscience is to find this link between neurophysiological activity and psychological activity, also called the neurobiological basis of behavior.^12^^,^^53^ Hence, researchers have defined a new concept, “neural efficiency,” to quantify the level of efficiency of collaborative effort of the brain in solving a difficult cognitive task (for a complete review see Ref. 54). Neural efficiency can be computed from physiological parameters such as heart rate variability, EEG measurements, and fMRI recordings, and recently from fNIRS findings.^55^^–^^57^ Researchers preferred to find a relationship between the neuropsychological data and neurophysiological data mostly in terms of correlation coefficients or regression analysis. The equation eventually obtained transforms one finding to another one, consequently assumes a causal relationship.

Borrowing an idea from neurophilosophy, one can impose the duality principle in brain’s operations where an independent relation between the brain and mind can be the source of cognition. Hence, one can simply propose a metric (an index) that combines these two so-called independent measures; namely, the behavioral findings with physiological findings in assessing the neurocognitive effort. Here, a new combined metric is proposed, by which NCR is defined as follows:

$$CQ_{i}=ACC_{i}/RT_{i},$$ (5)

$$R_{i}=GE_{i}^{CM}/GE_{i}^{DM},$$ (6)

$$NCR_{i}=CQ_{i}\times R_{i},$$ (7)

where i is the stimulus type, ACC is the accuracy in percentage, and RT is the reaction (response) time in seconds. CQ is defined for each stimulus type. $NCR_{i}$, as calculated from Eq. (7), can be assumed to be a biomarker specific for each subject group (i.e., healthy controls, patients with migraine, OCD, or schizophrenia disorder). The underlying assumption in proposing this index as a biomarker is that the *GE* of a default mode network should be different than the GE computed during a cognitive task ($GE^{CM}\neq GE^{DM}$) and that the ratio of the two [$R_{i}$ calculated as in Eq. (6)] can be considered as an objective indicator of attention and inhibition control. A reasonable expectation would be that $R_{i}\geq 1$ for healthy subjects. In fact, one can even hypothesize that an increased demand for inhibitory control can be associated with restructuring of the global network into a configuration that must be more optimized for specialized processing (functional segregation), more efficient at communicating the output of such processing across the network (functional integration), and more resilient to potential interruption (resilience). Thus, investigation of graph theoretical metrics under varying levels of inhibitory control can provide clinicians with a quantitative and objective metric in their clinical decision processes.^53^^,^^58^^,^^59^

## 3 Results

### 3.1 Behavioral Results

**Fig. 4:**
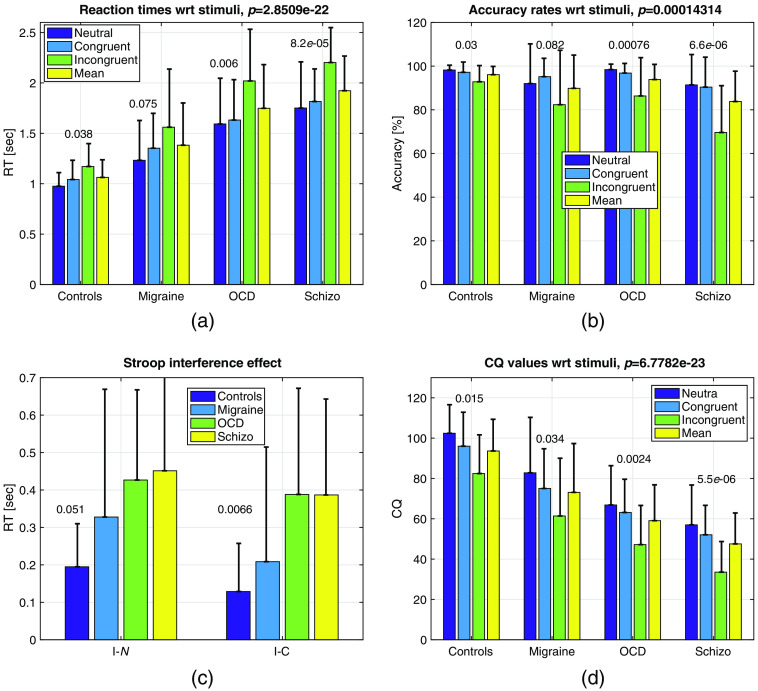
(a) RT values at the columns of Table 3 for all subjects across stimuli. p values are presented on top of bars. (b) ACC values at the columns of Table 4 for all subjects across stimuli. p values are presented on top of bars; (c) Stroop interference effect in reaction times; (d) CQ values computed by Eq. (5). Error bars represent the standard deviations. All values in these graphs are given in Tables 3–5.

The reaction times and accuracy rates for the subjects for all stimuli types are given in Figs. 4(a) and 4(b). Reaction times are calculated by averaging the response times to all the responses, not just the correct answers.

Two-way ANOVA for RT values yielded significant values for subject comparison ($p\ll 10^{-6}$), and stimulus type ($p\ll 10^{-6}$) and no interaction for SUB⋆STIM (p=0.749). Accuracy was calculated by taking the ratio of total number correct answers to total number of questions. Two-way ANOVA for the ACC yielded significant values for subject comparison ($p\ll 10^{-6}$), and stimulus type ($p\ll 10^{-6}$) and no interaction for SUB⋆STIM (p=0.1588).

CQ with respect to subjects and stimuli can be seen in Fig. 4(d). Two-way ANOVA for CQ yielded significant values for subject comparison ($p\ll 10^{-6}$), and stimulus type ($p\ll 10^{-6}$) and no interaction for SUB⋆STIM (p=0.9958). CQ can also be considered as a metric of cognitive load. In several studies, such scores from different tests are linearly combined (sometimes with weights) to provide a stronger metric.

### 3.2 fNIRS Data Analysis

#### 3.2.1 GE analysis

**Fig. 5:**
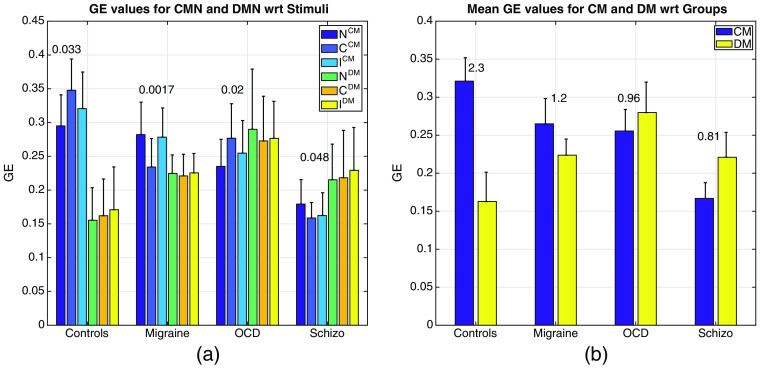
(a) $GE^{CM,DM}$ values for all subjects across three types of stimuli. p values are presented on top of bars, the error bars are the standard deviations and are also given in Tables 6 and 7. (b) Mean of $GE^{CM,DM}$ values for all stimuli with respect to subject group. The numbers above the bars are the ratios ($R_{i}=GE^{CM}/GE^{DM}$) as explained in Sec. 2.4. The error bars are the within group standard deviations.

The computation of $GE^{CM,DM}$ from the $FC^{CM,DM}$ matrices yielded quite interesting dynamics as shown in Figs. 5(a) and 5(b). First, PCA and Θ optimized $GE^{CM}$ values showed a strong statistically significant difference within subjects [see the p values displayed on top of the bars of Fig. 5(a)] with the optimal choice of principal components and Θ values given in Table 2. This is expected since the optimization for the PCA components and Θ is supposed to lead to statistically significant different $GE^{CM}$ values for each subject group. Group averaged $GE^{CM}$ values were higher for controls than for the patients [see Fig. 5(b)] although different optimized parameters were used for each subject group, whereas no significant difference was observed for any of the subject-wise $GE^{DM}$ values (p>0.05).

Second, both the $GE^{CM}$ and $GE^{DM}$ values were observed to be different between subject groups [as more evident from the means graph in Fig. 5(b)]. As the $GE^{CM}$ value decreases from healthy controls to schizophrenics, the $GE^{DM}$ increases. This is somewhat expected since the threshold node value for CM ($\Theta^{CM}$) that yielded strong significance ended up being lower in patients. *Vice versa*, the threshold node value for DM ($\Theta^{DM}$) were higher for patients than controls in most of the cases. Higher value of $GE^{CM}$ in controls over patients could mean that a healthy brain recruits a wider brain circuitry with more efficiency during a cognitive task, whereas diseased brain cannot. In contrast, higher values of $GE^{DM}$ for diseased population might be due to a domination of the DM leading to a lesser space for CM; hence, the poor performance on cognition related activities. Figure 5(b) shows the ratio of mean of $GE^{CM}/GE^{DM}$ for each subject group. The ratio is in favor of healthy controls and significantly lower in diseased groups.

A note of caution is that these values of $GE^{CM,DM}$ depend heavily on the choice of optimal principal components and threshold values when computing the $FC^{CM,DM}$ matrices. Hence, many iterations and heuristic reasoning were employed to find the best GE values that would yield the highest statistical significance for GE. An iterative approach in search of the best PCA components yielded the results in Table 2 that led to the highest statistical significance for the $GE_{i}^{CM}$, i=N,C,I for a specific group (i.e., controls, migraine, OCD, or schizophrenia subjects).

Similarly, the convergence to the optimal threshold values required an extensive search. The number of highest connectivity values had to be found specifically for each subject group that would lead to the most statistically significant p-value for the $GE^{CM}$. Table 2 reports the best combinations for PCA components to be used in the computation of the new $FC^{CM}$ matrices and the threshold values that would give the highest significance in the calculation of the $GE^{CM}$ and $GE^{DM}$ values.

**Fig. 6:**
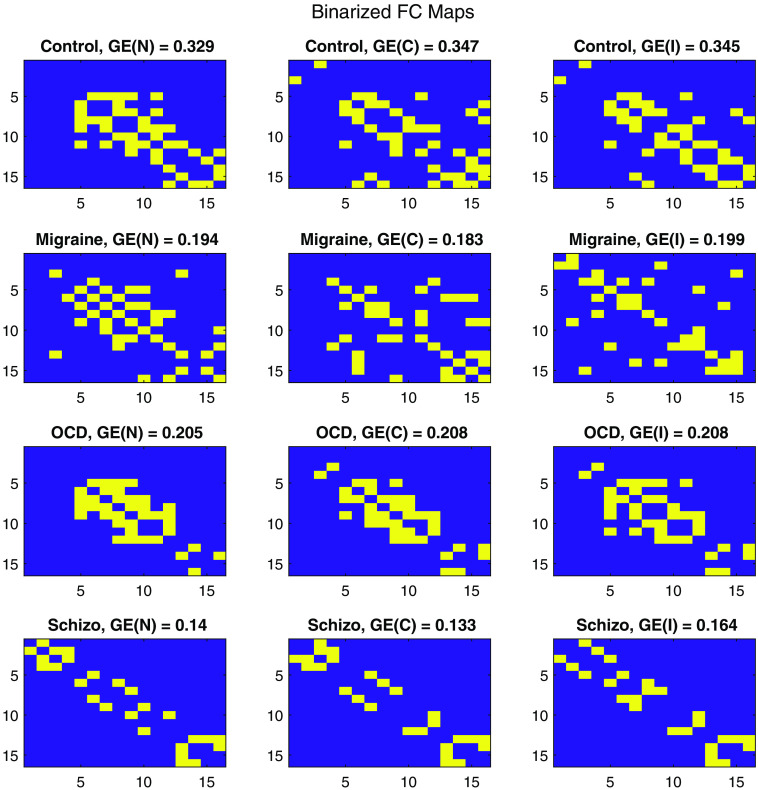
Averaged FC binary maps of subject groups (rows) with respect to stimulus types (columns) where the $GE^{CM}$ values are presented at the titles.

Figure 6 provides global representations for mean of $FC^{CM}$ maps, with the *GE* values printed on top of each connectivity map. These maps were obtained by averaging the PC-based FC(N,C,I) for subject groups and then reconstructing the $FC^{CM}(N,C,I)$ with the PCA components given in Table 2 to find an averaged representative $FC^{CM}(N,C,I)$. $GE^{CM}(N,C,I)$ were computed by thresholding for the highest number of entries given Table 2.

A group-wise representation of such binary matrices can be seen in Fig. 6. These representative binary FC matrices ($FC^{CM}$) were computed by first subject-wise averaging of $FC^{CM}$ ($\underset{s}{\sum }FC_{s}^{CM}$ where s is the subject number within a subject group) and then applying the thresholding approach as in Eq. 4 with the parameters in Table 2.

#### 3.2.2 NCR analysis

**Fig. 7:**
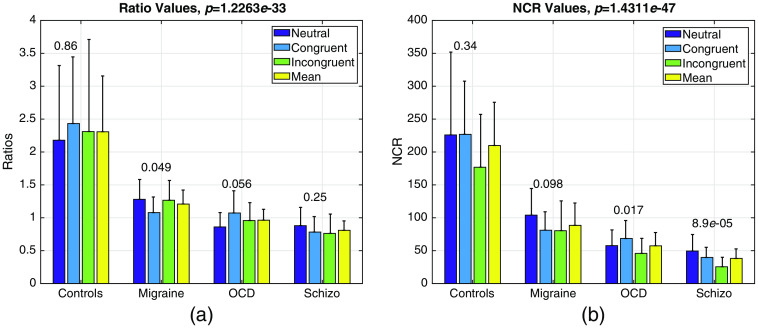
(a) R values as computed by Eq. (6) for all stimuli across subjects and (b) NCR values as computed by Eq. (7) for all subjects across stimuli all presented with their standard deviations as error bars. Within group p, values are presented on top of bars, whereas inter group p values are given at the title of the graphs.

Both the R and *NCR* values computed by Eqs. (6) and (7) elucidated strong statistical significance between healthy controls and rest of the diseased groups as seen in Figs. 7(a) and 7(b).

Two-way ANOVA for NCR yielded significant values for subject comparison ($p\ll 10^{-6}$), and stimulus type (p=0.001) and no interaction for SUB⋆STIM (p=0.3575). As expected *NCR* values are highest for the healthy controls since both the *CQ* and R values are higher in controls.

### 3.3 Receiver Operating Characteristic Analysis

**Fig. 8:**
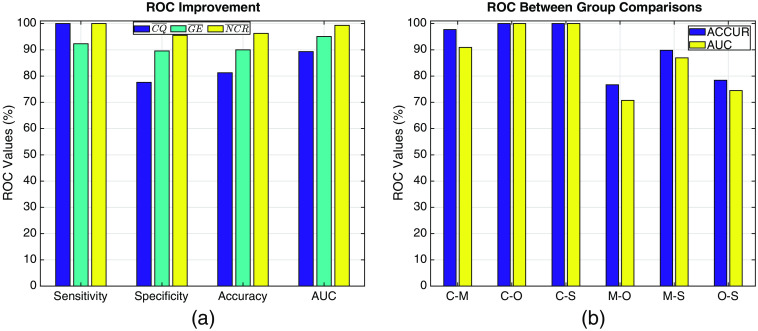
(a) ROC values computed for mean values across stimulus types of $\bar{CQ}$, ${\bar{GE}}^{CM}$, and $\bar{NCR}$ between healthy controls and the rest of the patients. SENS: sensitivity, SPEC: specificity, ACCUR: accuracy, AUC: area under the curve. (b) ROC values between pairs of subject groups. C: controls, M: migraineurs, O: OCD patients, S: schizophrenia patients.

Receiver operating characteristics (ROCs) provide a comparison of the accuracy of classification between the healthy controls and the rest of the cases (2-case comparison). Figure 8(a) shows the results of performance of classification by using means across three stimulus types of the various different parameters obtained in this paper.

As can be observed from Table 8, the accuracy of classification with respect to mean of *CQ* ($\overset{¯}{CQ}=1/3\underset{i}{\sum }CQ_{i}$, i=N,C,I) values is 81.2% while the accuracy with respect to mean value of *NCR* ($\bar{NCR}$) peaks at 96.3% with a very high AUC score of 99.33%.

**Table 3:** Reaction times in milliseconds (mean ± standard deviation). Number of subjects are given in parentheses.

Group	NS	CS	ICS	p-value
Controls	975.7±134.7	1041.8±190.8	1170.6±227.6	$0.0375^{*}$
Migraineurs	1233.0±394.6	1352.2±345.9	1560.7±576.8	0.0748
OCD	1593.1±453.3	1631.5±400.7	2019.7±512.7	$0.006^{*}$
Schizo	1750.9±458.1	1815.5±323.4	2202.2±346.4	$<0.0001^{*}$
p-value	$<10^{-7}$	$<10^{-9}$	$<10^{-9}$	0

As promised, the ROC values show a dramatic increase once the features derived from fNIRS findings are included alongside the behavioral findings. ROC values computed from the $\bar{NCR}$ holds a great promise. Except the sensitivity, there are remarkable increases in the other ROC parameters between $\overset{¯}{CQ}$ and $\bar{NCR}$. Specificity increased by 17.9%, accuracy by 15.1%, and AUC by 10% as seen in Table 8. One might wonder how the classification performance of $\bar{NCR}$ behaves between non-healthy subjects. That ROC analysis is given in Fig. 8(b). AUC values for controls versus diseased patients are very high; hence, the sensitivity and specificity of the $\bar{NCR}$ values are very promising in separating healthy from non-healthy brain. The classification accuracy of $\bar{NCR}$ between controls and OCD patients is 100% but it drops to 74.47% between OCD and schizophrenia patients. The accuracy is high at 86.95% between migraine and schizophrenia patients.

## 4 Discussions

### 4.1 Behavioral Findings

The Stroop task is one of the most favorable neuropsychological tests to investigate the cognitive impairments in attention and inhibition control.^31^^,^^60^^–^^62^ The behavioral results in this paper confirm the claim that both the reaction times and accuracy rates are statistically different between healthy controls and patients with neuropsychiatric diseases as seen in Tables 3 and 4 and in Figs. 4(a)–4(d). The reaction rates for a specific stimulus type increase, accuracy rates decrease (error rates increase) as the severity of the attention and inhibition controls are impaired. This phenomenon has been also observed in this study as seen in Figs. 4(a)–4(d). In a review by Foti et al.,^63^ several studies reported an impairment in executive functions observed in migraine patients as measured by different neuropsychological tasks, including the Stroop task. The Stroop interference effect, as measured by the difference in the reaction times, provides an insight to inhibition (I-N) and facilitation (I-C) controls.^31^ The results are in parallel with most of the findings in literature where an impairment in executive functions in neuropsychiatric patients was observed for many tasks including the Stroop task. There are several methods to further quantify the behavioral results of the stroop test.^62^ These are usually in form of combinations of error (accuracy) rates and reaction times. This paper used a simpler metric: CQ, where the difference in executive functions of these four groups were emphasized better than any one parameter alone. In fact the classification accuracy of this metric between healthy controls and diseased subjects was 76.25% as seen in Fig. 8(a). In a study by Erdodi et al.,^60^ classification accuracy of inverted Stroop test metrics between healthy controls and patients that were clinically referred for neuropsychological assessment were found to be less sensitive (14% to 25%), but comparably specific (85% to 90%) while the findings in this study were contradictory with very high sensitivity (100%) but less specificity (77.6%) for this metric. Certainly there are many differences especially in the choice of subject groups, the Stroop test employed and the parameters used in the analysis of that study and this one, but it is evident that the behavioral parameters alone cannot yield high accuracies in classification for neuropsychological assessment.

### 4.2 fNIRS Findings

GE has been proposed and shown to be a robust and reliable biomarker in many fNIRS studies.^21^^,^^64^^–^^69^ In most cases resting fNIRS data were used to calculate the FC matrices. GE values were always computed after a threshold value was adapted. This threshold value was an actual correlation coefficient value. Since a fixed correlation coefficient threshold would yield different number of non-zero entires in the binarized FC matrices, the GE values would be incomparable (For a good review on the appropriate choice of a threshold value, we can refer to Ref. 70). Regardless of the choice of PCA components and threshold values, almost all of the studies concluded that as the cognitive impairment increases, global network efficiency decreases compared to healthy controls. GE is also shown to be affected by age.^64^^,^^71^ The GE based findings in this paper are in alignment with the literature, where a decline in GE was observed for neuropsychiatric patients. On average across all stimulus types, there was a 17% reduction in the $GE^{CM}$ of migraineurs, 20% of OCD, and 48% of schizophrenics from controls as can be inferred from Table 6.

### 4.3 On the Classification Accuracy of NCR

There have been several studies that investigated the classifier accuracy for schizophrenia patients.^72^^–^^78^ Yet, the same cannot be said for migraine and OCD patients. Table 9 is a selection of such studies where search words: {fNIRS, classification, schizophrenia, and accuracy} were used.

Most of the classification studies including schizophrenia patients reported a classification accuracy in the range of 76% to 89.7% as seen in Table 9. This study achieves at a 100% accuracy score for schizophrenia patients as seen in Fig. 8(a). The strength of this value is inherent to the computation of the $\bar{NCR}$ where behavioral and physiologic data are fused. The accuracy is lower between neuropsychiatric patients as seen in Fig. 8(b) columns M-O, M-S and O-S with M-S being the highest at 89.81%. This is an indicator that cognitive impairments in migraine patients might not be as severe as the OCD and schizophrenics, which are close to dissociative disorder diseases.

**Table 4:** Accuracy rates in percentages (mean ± standard deviation).

Group	NS	CS	ICS	p-value
Controls (13)	98.2±2.2	97.2±4.7	92.8±7.4	$0.0297^{*}$
Migraineurs	92.0±18.2	95.2±8.4	82.3±24.9	0.0815
OCD	98.4±2.5	96.8±4.4	86.3±17.5	$<0.001^{*}$
Schizo	91.4±13.9	90.4±13.7	69.6±21.4	$<0.00001^{*}$
p-value	$<10^{-7}$	$<10^{-9}$	$<10^{-8}$	0

### 4.4 Proposal

This study is yet another one that proposes an algorithmic approach to the data analysis pipeline of fNIRS studies. The aim is to improve the clinical significance of the features extracted from fNIRS recordings so as to pledge an everlasting position of fNIRS in clinical settings. Only a handful studies investigated the differential diagnostic accuracy of fNIRS features.^85^^–^^89^ To start-off, here is a checklist of specific expectations of any fNIRS-based algorithmic approach for a clinical study:

**E1:** Provide clinically relevant information regarding brain physiology
**E2:** Provide strong specificity for clinical data
**E3:** Provide a better statistics than behavioral data alone
**E4:** Provide an easy and applicable/adoptable algorithm

fNIRS has been one of the few instruments that can provide insight to brain neurophysiology non-invasively and rapidly. Yet, these two offerings should match with the expectations listed above. Since fNIRS provides information regarding the cerebrovascular reactivity to cognitive or physiological stimuli, we expect that any measurement from patients with brain disorders should provide insight to neurobiology of the disease.

To address **E1**, fNIRS is famous for bestowing local hemodynamic activity. Moreover, *GE* extracted from the dynamic changes of such a local data elucidate the level of collaborative effort exerted during a cognitive task. So with a number such as *GE* one can capture the hemodynamics of cognition.

To address **E2**, several groups reported medium to high accuracies for classification of fNIRS signals.^29^^,^^34^^,^^73^^,^^74^^,^^79^^,^^81^ These studies mostly included two groups: healthy controls and a diseased group. No multi-group comparison has been attempted with fNIRS, unlike fMRI.^34^ *NCR* derived from CQ and ratio of GEs are shown to be highly specific for diseases.

To address **E3**, so far the statistical significance of behavioral data have been praised in many studies in classifying patient groups. fNIRS is expected to improve the statistics and this is what NCR offers. It gives a higher accuracy in separating healthy from diseased brain, much like a blood pressure monitor.

To address **E4**, a valid critique is that the proposed approach is easy and applicable. It is more of an iterative approach than a theoretical one. Yet, the ${\mathrm{FC}\text{-}(\mathrm{PC})}^{2}$ seems to do the trick in separating the FC matrices.

Interestingly there are not many studies of other psychiatric diseases. Still there are pioneering works on autism spectrum disorder,^14^ obsessive compulsive disorder,^10^ depression, and migraine.^23^ There is always those who have not lost faith in fNIRS.^11^^,^^12^ A very hopeful study by Ehlis et al.^9^ points us to the right direction: future studies should also focus on the usefulness of fNIRS as a supportive tool for choosing the most promising treatment approach for a specific patient. Using fNIRS, neurophysiological markers that might predict treatment outcomes (and may thus be relevant for personalized medicine) could be easily identified.^9^ Several studies actually achieved this ambitious goal set by Ehlis et al. For a good review of use of fNIRS in psychiatry, please see Refs. 9, 90, and 91, specifically in autism,^14^ and its role in neurofeedback,^92^ in pain,^93^ and in neurology.^94^ Only a handful of them are listed in Table 9. This study is the first in several aspects: (1) to show a high specificity of fNIRS for various types of neuropsychiatric diseases (more than 2); (2) in providing an fNIRS-derived biomarker (namely the *NCR*) with very high accuracy that is also clinically relevant; and (3) in that it does not attempt to find a correlation between behavioral data and physiological data, rather it combines them since behavior cannot be produced without physiological activation. As observed by James, “A science of the relations of mind and brain must show how the elementary ingredients of the former correspond to the elementary functions of the latter.”^95^

## 5 Conclusion

This study is an extension of a previous work, which concluded that a PC-based approach should be preferred when generating the FC matrices. Separating the FC into a CM and DM network led to the ratio of the GE values calculated from these two matrices. This ratio was then multiplied with the CQ, which is a direct measure of cognitive load. Therefore, a new biomarker, NCR, was generated and proposed. The mean NCR across all stimuli for four subject groups in Table 10 ($\overset{¯}{NCR}(\text{Control})=210\pm 66$, $\overset{¯}{NCR}(\text{Migraine})=89\pm 34$, $\bar{NCR}(\mathrm{OCD})=57\pm 20$, and $\overset{¯}{NCR}(\text{Schizo})=38\pm 15$, $p<10^{-10}$) gives the best classification accuracy with respect to ROC between healthy controls and diseased subjects (ACCUR=96.25%, and AUC=99.31%), much better than the accuracies obtained from only CQ, behavioral parameter ($\overset{¯}{CQ}(\text{Control})=94\pm 16$, $\overset{¯}{CQ}(\text{Migraine})=73\pm 24$, $\bar{CQ}(\mathrm{OCD})=59\pm 18$, and $\bar{CQ}(Schizo)=48\pm 15$, $p<10^{-8}$) where (ACCUR=81.2%, and AUC=89.3%). The results are all in favor of this biomarker. So we might conclude that fNIRS-derived NCR is a strong candidate as a biomarker for neuropsychiatric diseases. It can safely be used in diagnosis and prognosis of neuropsychological assessments of at least a group of neuropsychiatric disorders.

## 6 Appendix

The following tables present the values of the figures in the manuscript.

### 6.1 Behavioral Data

The following Table 5 is formed from Tables 3 and 4.

**Table 5:** CQ values (mean ± standard deviation).

Group	NS	CS	ICS	p-value
Controls	102.42±14.15	96.00±16.87	82.44±19.19	$0.0147^{*}$
Migraineurs	82.80±27.50	75.04±19.68	61.41±28.62	$0.0341^{*}$
OCD	66.85±19.49	63.12±16.49	47.18±19.44	$0.0024^{*}$
Schizo	56.99±19.77	52.05±14.62	33.53±15.19	$<0.00001^{*}$
p-value	$<10^{-7}$	$<10^{-9}$	$<10^{-8}$	$<10^{-8}$

**Table 6:** Optimized $GE^{CM}$ values computed from $FC^{CM}s$ (mean ± standard deviation).

Group	NS	CS	ICS	p-value
Controls	0.2951±0.0482	0.3478±0.0543	0.3207±0.0634	0.0326
Migraineurs	0.2821±0.0275	0.2342±0.0321	0.2784±0.0287	0.0017
OCD	0.2350±0.0662	0.2768±0.0549	0.2548±0.0549	0.0197
Schizo	0.1793±0.0527	0.1587±0.0701	0.1624±0.0635	0.0482
p-value	$<10^{-10}$	$<10^{-10}$	$<10^{-10}$	$<10^{-10}$

**Table 7:** Optimized $GE^{DM}$ values computed from $FC^{DM}s$ (mean ± standard deviation).

Group	NS	CS	ICS	p-value
Controls	0.1554±0.0482	0.1621±0.0543	0.1710±0.0634	0.78
Migraineurs	0.2246±0.0275	0.2210±0.0321	0.2255±0.0287	0.82
OCD	0.2901±0.0891	0.2728±0.0662	0.2766±0.0549	0.71
Schizo	0.2153±0.0527	0.2183±0.0701	0.2292±0.0635	0.70
p-value	$<10^{-4}$	$<10^{-4}$	$<10^{-4}$	$<10^{-10}$

### 6.2 GE Values

**Table 8:** ROC analysis of mean values of features. SENS: sensitivity, SPEC: specificity, ACCUR: accuracy, AUC: area under the curve.

Feature	SENS	SPEC	ACCUR	AUC
$\bar{CQ}$	100	77.6	81.2	89.3
$\bar{GE}$	92.3	89.6	90	95.1
$\bar{NCR}$	100	95.5	96.3	99.3

**Table 9:** Classification performances of fNIRS studies.

Study	Diseased group	Features	Classifier	Accuracy
Chen et al.^72^	Schizophrenia	GLM based	SVM	85%
Dadgostar et al.^75^	Schizophrenia	Time series	SVM	87%
Einolou et al.^29^	Schizophrenia	Energy	SVM	84%
Eken et al.^79^	SSD	FC based	SVM	82%
Hahn et al.^78^	Schizophrenia	Time series	LOOCV	76%
Ji et al.^73^	Schizophrenia	FC based	SVM	89.7%
Shoushtarian et al.^80^	Chronic tinnitus	FC based	Naïve Bayes	78.3%
Xu et al.^81^	ASD	Time series	CNN & LSTM	93.3%
Yang et al.^74^	Schizophrenia	FC based	SVM	84.67%
Yang et al.^82^	MCI	Time series	CNN	98.61%
Yang et al.^83^	MCI	FC based	CNN	95.81%
Yoo et al.^84^	MCI	Time series	SVM	79.49%

### 6.3 NCR Values

**Table 10:** $\bar{NCR}$ values computed from Table 11 (mean ± standard deviation).

Group	$\bar{NCR}$
Controls	209.89±65.67
Migraine	88.50±33.97
OCD	57.36±20.28
Schizo	38.22±14.52
p-value	$<10^{-10}$

**Table 11:** *NCR* values computed from Eq. (7) (mean ± standard deviation).

Group	NS	CS	ICS	p-value
Controls	225.97±125.93	226.84±80.88	176.85±80.35	0.342
Migraine	104.00±40.53	81.09±28.04	80.41±45.20	0.098
OCD	57.68±23.92	68.54±27.39	45.88±22.78	0.017
Schizo	49.48±25.05	39.68±15.37	25.48±14.43	$<10^{-5}$
p-value	$<10^{-10}$	$<10^{-10}$	$<10^{-10}$	$<10^{-10}$
